# Benign Acute Childhood Myositis in a Pediatric Patient Post Influenza B Infection

**DOI:** 10.7759/cureus.51171

**Published:** 2023-12-27

**Authors:** Michael C Huzior, Brendan P Chernicki, Lisa Nguyen, Bobby Kumar

**Affiliations:** 1 Pediatrics, Nova Southeastern University Dr. Kiran C. Patel College of Osteopathic Medicine, Davie, USA; 2 Pediatrics, Salah Foundation Children’s Hospital at Broward Health Medical Center, Fort Lauderdale, USA

**Keywords:** pediatrics medicine, pediatrics education, msk symptoms, viral myositis, inpatient pediatrics

## Abstract

Benign acute childhood myositis (BACM) is a mild and self-limited sudden onset of lower extremity pain during or following recovery from a viral illness. It is characterized by difficulty walking due to severe bilateral calf pain, which usually resolves in three days. It is typically appreciated during times of large influenza outbreaks and epidemics. The most severe complication can be rhabdomyolysis without proper treatment and can lead to renal damage and potential renal failure. There are limited reported cases of BACM and therefore no clear guidelines in the treatment or management of the condition. This case is unique in the sense that the patient had leg pain the entire month prior to presentation in the absence of trauma or injury, and it is believed that the pre-existing myalgia may have been exacerbated by an upper respiratory infection (URI) that started a few weeks after the leg pain onset. In addition, this patient's creatine kinase peaked at over 13,000 U/L, which is three to five times higher than the average of other reported children with this condition. The patient is a five-year-old male who presented to the emergency department with bilateral leg pain and difficulty ambulating. His guardian reported that the leg pain began one month prior to presentation and worsened to the point where he could no longer ambulate, following a fever and cough that began one week prior to presentation. A respiratory viral panel was positive for influenza B, and initial creatine kinase (CK) levels were greater than 10,000. A diagnosis of BACM was made, and supportive care was initiated. BACM is an infrequent complication following a viral infection that is typically treatable with hydration management and routine CK monitoring. Symptoms of BACM are usually limited to muscle pain and weakness, but it can progress to rhabdomyolysis and renal failure if not managed properly. It is therefore crucial that physicians monitor CK values daily until a downtrend is noticed and symptoms begin to resolve.

## Introduction

Virus-induced myositis, also known as a benign acute childhood myositis (BACM), is a rarely reported complication that can present after a viral respiratory infection [1]. The incidence of this complication is not currently known, but the most reported cases involve school-aged children, with a median age of 8.3 years old and male-to-female ratio of 2:1 [2]. Several authors have confirmed influenza B with association of viral myositis, but it has also been associated with influenza A, adenovirus, parainfluenza, coxsackievirus, and *Mycoplasma pneumoniae* [3]. The condition often starts with prodromal viral symptoms, such as headache, fever, rhinosinusitis, cough, and fatigue. A few days following, patients then describe symmetric leg pain that lasts on average up to three days [1]. There are currently no specific guidelines or recommended steps of management for BACM as the condition is mostly self-limiting with the exception of the rare complication of rhabdomyolysis, which would require further workup and management [1]. Common practice in BACM management involves trending creatine kinase (CK) values during a hospital stay as a primary measure of the patient’s inflammatory response. Reports have suggested that CK values do not necessarily correlate with the severity of symptoms, but many physicians rely on CK values as a benchmark for discharge, wanting to ensure that renal function is preserved and there is no onset of myoglobinuria [4]. Patients who progress to rhabdomyolysis experience surge in CK levels and decline in kidney function.

## Case presentation

The patient is a five-year-old male patient with no significant past medical history who presented with bilateral leg pain. He was initially accompanied by his brother at bedside who contributed as a historian. The brother reported that the patient had been complaining of leg pain for roughly one month, and over the past few days, the pain worsened to the point where he could not ambulate independently. His brother also reported that about one week ago, the patient was having upper respiratory symptoms and fevers at home, which resolved on their own after a few days. Otherwise, the patient had no vomiting, diarrhea, or rashes. There were no other sick contacts in the household. Upon presenting to the ER, the patient was afebrile with stable vital signs. He was noted to have trouble standing and could not ambulate. Lab work revealed CK that remarkably elevated at 10,318 U/L, and otherwise creatinine within normal limits of 0.6 mg/dL and blood urea nitrogen (BUN) of 7 mg/dL. White blood cell (WBC) and hemoglobin were within normal limits at 4.96 k/uL and 12.9 g/dl, respectively. A respiratory viral panel was performed given his history of recent fever and upper respiratory symptoms, which was positive for influenza B. The combined elevated CK, patient presentation, and history of upper respiratory infection were consistent with benign acute childhood myositis. To rule out rhabdomyolysis, a urine analysis was done, which revealed no blood, trace protein, and a normal specific gravity of 1.022. He was given two normal saline boluses while in the ER and then admitted to the pediatrics inpatient unit for continuation of IV fluids and monitoring. 

Lactated ringers were administered at a rate of maintenance x1.5, and the patient's CK level was trended until it reached an acceptable level for discharge. Adequate hydration was also encouraged. The patient did not present with upper respiratory symptoms or fever despite his influenza B diagnosis, and it was therefore decided to not begin with Tamiflu treatment but rather monitor for any worsening symptoms. Over the next day, bilateral leg pain, limited bilateral dorsiflexion of the feet, and an abnormal gait were still present on physical examination. Physical therapy and active stretching were recommended for improvement of the poor dorsiflexion. 

**Figure 1:**
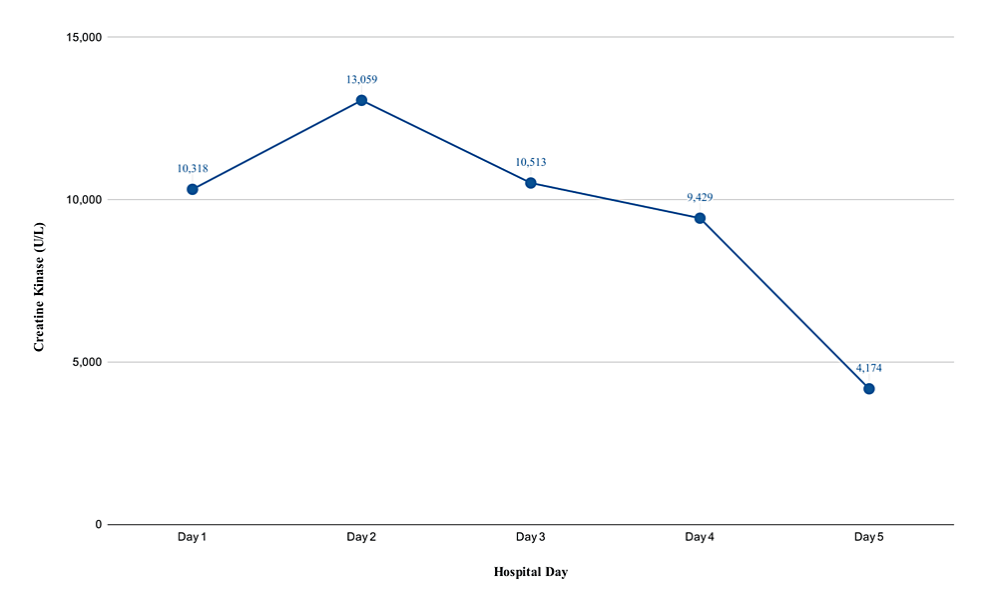
Creatine kinase levels during hospital course

As demonstrated in Figure 1, CK levels continued to rise, peaking on day 2 at 13,059 U/L, but then it began to trend downwards, measuring 10,513 U/L, 9,429 U/L, and 4,174 U/L, on days 3, 4, and 5, respectively. By day 5, the CK value dropped by more than half the peak recorded value and musculoskeletal (MSK) symptoms began to improve. The patient was then discharged, and the family was advised to provide adequate hydration and Tylenol as needed for pain. The family was also instructed to be vigilant for worsening leg pain, difficulty ambulating, blood in urine, and upper respiratory compromise.

## Discussion

This patient's hospital sequelae matches other case reports on BACM in that the patient is a male presenting with bilateral leg pain following an influenza B infection. The exact mechanism behind viral myositis due to influenza B infection is not fully understood. However, there have been animal studies that have shown that mice inoculated with influenza B virus via intramuscular injection into the quadriceps developed elevated creatinine phosphokinase on days 1 and 2 post-inoculation and mononuclear inflammation in perimysial connective tissue on days 2 and 3 post-inoculation [5]. The cellular mechanism postulated for the muscle damage that leads to viral myositis and subsequent rhabdomyolysis is the virus enters the muscle cells and damages the plasma membrane, causing extracellular calcium to rush into the muscle cells, leading to excessive contraction and depleting ATP levels. The depletion of adenosine triphosphate (ATP) leads to buildup of calcium without subsequent removal by Ca2+ ATPase, leading to activation of proteases and phospholipases that damage the plasma membrane and mitochondria, leading to muscle necrosis and apoptosis [6]. Rhabdomyolysis can be distinguished from viral myositis due to its extremely elevated CK levels of usually 100,000, presence of myoglobinuria and hematuria, and potential for acute kidney injury. The mechanism of acute kidney injury in rhabdomyolysis is unknown, but evidence suggests intrarenal vasoconstriction, direct and ischemic tubule injury, and tubular obstruction from leakage of cell contents [6-8].

Upon admission, the patient struggled with ambulation, and he continued to have difficulty walking for the following two days of his hospital stay. Of reported cases of BACM, the average CK level falls between 1,400 and 4,000 U/L, but our patient had levels surpassing 13,000 U/L with no worsening of his initial symptoms [2]. There are limited data on the management and expectations of children who develop this complication, but it should be noted that CK levels may continue to rise throughout the hospital stay despite continuation of lactated ringer fluid management, the accepted treatment for BACM. This is likely due to symptom onset occurring with the initial rise of CK, which may be captured on day 1 of hospital admission. The management of this patient included the trending CK values for five days and monitoring for the presence of myoglobinuria. Following the precedent of published cases of BACM, this patient was discharged when CK levels began to trend downward, MSK symptoms began to improve, and there was no sign of myoglobinuria that would indicate the onset of rhabdomyolysis. Physicians should use these parameters in addition to clinical judgment when determining the appropriate time to discharge a patient after a documented BACM event. Because BACM is self-limiting and carries an excellent prognosis, there are no formal recommendations to guide the treatment and discharge process. Patients should expect to see an improvement of symptoms with the use of hydrating fluids, analgesics, and bed rest [3].

## Conclusions

BACM is a self-limiting condition seen in young males under the age of six after the onset of an acute respiratory infection, most commonly in the setting of influenza B. Patients may report symmetrical leg pain that may manifest in the inability to ambulate for several days. The clinical course of this condition should be monitored by trending CK values while remaining vigilant for progression to rhabdomyolysis, which may be evidenced by the presence of myoglobinuria and increasing levels of CK. Clinicians should also expect an initial rise in CK values, especially if hospital admission coincided with the acute onset of symptoms, with the expectations that the values should drop over the following days. Downtrending CK values, improvement in symptoms, and absence of rhabdomyolysis are positive indicators of BACM resolvement, but clinicians should use clinical judgment in evaluating the patient before permitting discharge.
